# Iris metastasis as the initial presentation of metastatic esophageal cancer diagnosed by fine needle aspiration biopsy: A case report

**DOI:** 10.1097/MD.0000000000026232

**Published:** 2021-06-04

**Authors:** Hiroko Ozawa, Yoshihiko Usui, Yoji Takano, Naoki Horiuchi, Tohru Kuribayashi, Toshihide Kurihara, Lois E.H. Smith, Kazuo Tsubota, Yohei Tomita

**Affiliations:** a Department of Ophthalmology, Kawasaki Municipal Hospital, Kanagawa; b Department of Ophthalmology, Keio University School of Medicine; c Department of Ophthalmology, Tokyo Medical University, Tokyo; d Department of Ophthalmology, Kawasaki Municipal Ida Hospital; e Department of Radiation Oncology, Kawasaki Municipal Hospital, Kanagawa, Japan; f Department of Ophthalmology, Boston Children's Hospital, Harvard Medical School, Boston, MA; g Tsubota Laboratory, Inc., Tokyo, Japan.

**Keywords:** esophageal cancer, fine needle aspiration biopsy, iris metastasis, radiotherapy, secondary glaucoma, squamous cell carcinoma

## Abstract

**Rationale:**

Metastasis of neoplasms to the eye is quite uncommon. In this case report, we describe a patient where primary esophageal cancer was diagnosed by fine needle aspiration biopsy (FNAB) of an iris tumor.

**Patient concerns:**

A 70-year-old male complained of redness and discomfort in the right eye.

**Diagnosis and interventions:**

The patient's right eye was diagnosed as idiopathic uveitis, and a topical steroid was administered. As vitreous opacities were observed even after topical therapy, oral prednisolone was administered. On slit-lamp examination of the right eye, an iris mass with neovascularization was seen in the anterior chamber. A metastatic tumor was suspected, and FNAB was performed. Histology revealed squamous cell carcinoma. Systemic workup revealed esophageal cancer with several metastases. Best-corrected visual acuity decreased to 20/400, and intraocular pressure was 40 mmHg in the right eye. Two iris tumors with neovascularization were present extending into the anterior chamber with posterior iris synechiae and 360 degree peripheral anterior synechiae. Intraocular pressure in the right eye was medically managed with hypotensive eye drops and oral acetazolamide. Iris metastases were treated with 40 Gray of radiation therapy and concurrent chemotherapy.

**Outcomes:**

The tumor regressed, but intraocular pressure was refractory to treatment because of 360 degree goniosynechial closure. The right eye lost light perception six months after treatment commenced, and the patient died 9 months after the onset of therapy due to multiple systemic metastases.

**Lessons:**

This is a rare case of masquerade syndrome without systemic symptoms in which FNAB of an iris tumor led to a diagnosis of metastatic esophageal squamous cell carcinoma. Although the patient lost his sight due to uncontrollable ocular hypertension, systemic chemotherapy, and radiation therapy were initially effective in the treatment of the metastatic iris tumor. As the prognosis of patients with metastatic iris tumors is poor, it is important for ophthalmologists to consider such diagnoses and conduct systemic investigations when necessary.

## 1 Introduction

Metastasis of neoplasms to the eye is quite uncommon.^[1]^ It is often difficult to diagnose the disease in the early stages because of masquerade syndrome. Masquerade syndrome is an ophthalmic disease with inflammation (often a malignant neoplasm) that mimics chronic idiopathic uveitis. The morbidity of masquerade syndromes among patients with uveitis was reported as 5%.^[2]^ Many disorders, including malignant lymphoma, leukemia, metastatic tumors, uveal melanoma, juvenile xanthogranuloma, and retinoblastoma, may masquerade as idiopathic uveitis. Forty-eight percent of all masquerade syndrome is reported to be caused by malignant tumors, but only 2.5% of masquerade syndrome arises from metastatic malignant tumors.^[2]^ Masquerade syndrome is often treated as chronic idiopathic uveitis, and so the diagnosis and treatment of the malignant tumor itself are often delayed, negatively influencing the prognosis. Furthermore, iris metastases rarely occur, as 88% of ocular metastatic tumors are in the choroid, with just 9% in the iris^[3]^ which has abundant muscular tissue and less blood flow than the choroid.^[4]^ We report a case of an iris tumor metastasized from esophageal carcinoma which was initially treated as idiopathic uveitis, then diagnosed as metastasis by fine needle aspiration biopsy (FNAB) from the anterior chamber.

## 2 Case report

Ethical approval was not necessary for this manuscript because it was a case report. The patient has died, but his wife consented to the publication of this case. Written consent to publish was obtained.

**Figure 1:**
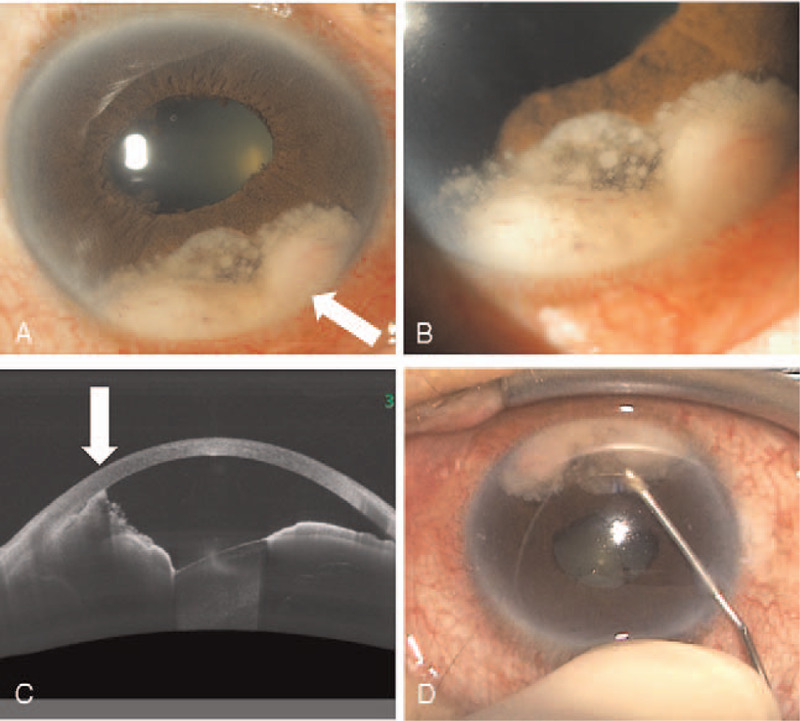
The anterior chamber images of iris tumor. (A) An image of the anterior segment of the right eye. There was a tumor located on the iris from 4 to 7 o’clock (arrow). (B) A magnified image of Figure 1A. Neovascularization occurred in the tumor. (C) An image of anterior segment optical coherence tomography. The angle was partially occupied by the tumor (arrow). (D) The technique of the fine-needle aspiration biopsy for iris tumors.

A 70-year-old male complained of redness and discomfort of the right eye (OD) and visited a local eye clinic. His medical history included hypertension, hyperlipidemia, hyperuricemia, and fracture of a left rib. Two weeks later, he was referred to a university hospital and diagnosed with anterior uveitis and scleritis of the right eye. Eye drops, including Tropicamide/Phenylephrine Hydrochloride, (Sandol P Ophthalmic Solution; Nihon Tenganyaku Kenkyusho Co., Ltd., Japan) and Neomycin/Betamethasone sodium phosphate (Rinderon-A, Shionogi & Co., Ltd., Japan) were started, and Dexamethasone sodium phosphate (Decadron Phosphate Injection, Aspen Japan K.K., Japan) subconjunctival injection was also administered. Blood samples and chest x-ray showed no obvious abnormalities related to uveitis. Two weeks later, vitreous opacity was detected, and 30 mg/day oral prednisolone (Predonine Tablets, Shionogi & Co., Ltd., Japan) was added. The intraocular pressure (IOP) of the right eye increased following the commencement of oral steroid treatment, and topical 0.5% Timolol Maleate twice a day (Timoptol ophthalmic solution; Santen Pharmaceutical Co., Ltd. Japan) was also started. Intraocular pressure returned to normal levels on tapering the steroid eye drops, and Timolol Maleate was discontinued. Two months after the initial treatment, best-corrected visual acuity (BCVA) had deteriorated, and the patient was referred to another hospital. At this point, his BCVA was 20/50 (OD) and 20/20 in the left eye (OS). IOP of both eyes were within normal limits. Slit-lamp examination detected conjunctival hyperemia and cells in his right anterior chamber. In addition, a yellowish-white mass with neovascularization was seen from 4 to 7 o’clock in the anterior chamber, and posterior iris synechiae were also detected (Fig. 1A and B). Cataract and vitreous cells were also observed. Anterior segment optical coherence tomography showed that the angle was partially closed by the mass (Fig. 1C).

**Figure 2:**
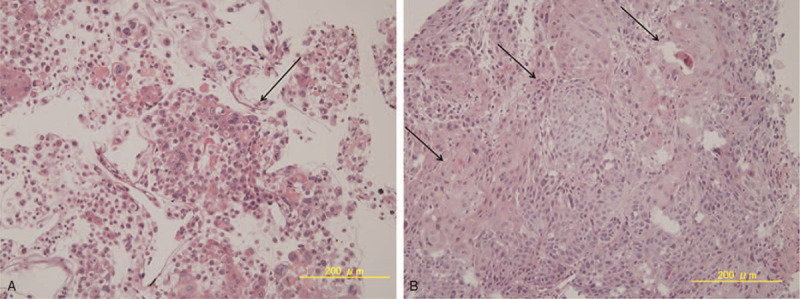
Histopathology and immunohistochemistry of biopsied iris tumor. (A) Squamous cell carcinoma (arrow) was identified in the tumor. The tumor cells are distributed as single cells or as small cellular aggregates. Atypical epithelial cells varied from eosinophilic to amphophilic and showed a polygonal shape with abundant cytoplasm. Keratinization was prominent, and the borders of cells were clearly identified, with intercellular bridges. In the atypical epithelial cells, the nuclear shape was oval to irregular, in which nuclear bodies were prominent, and there was increased chromatin. The size of the nucleus varied, and some cells had multiple nuclei (arrow, hematoxylin-eosin stain, ×200). (B) Histopathology of the biopsied esophagus. Tumor cells with an alveolar configuration proliferated profusely (arrow), in which cell division and amorphous nuclei and abundant cytoplasm were observed (hematoxylin-eosin, ×200).

The yellowish-white mass with neovascularization was suspected to be a metastatic iris tumor, and FNAB was performed as previously described.^[5]^ Briefly, under local anesthesia using 4% xylocaine, a 27-gauge needle attached to a 2.5 mL syringe entered the anterior chamber through a limbal site and aspirated the tumor located at 6 o’clock (Fig. 1D, see Video, Supplemental Video [The video that demonstrates fine-needle aspiration biopsy technique of the current case, 22 seconds, 5.3 MB.], which demonstrates this in more detail). Then the anterior chamber was refilled using balanced salt solution to normalize the IOP. The iris biopsy demonstrated atypical epithelial cells from eosinophilic to amphophilic, having a multi ridge, abundant cytoplasm, and keratinized in several sections. The boundary of cells was clear with intercellular bridges. Atypical epithelial cells had anisokaryosis and abundant mitosis, and their forms were irregular (Fig. 2A). With immunostaining, this tumor was diagnosed as squamous cell carcinoma.

As the iris tumor was likely to be a metastasis, systemic evaluations were initiated. Multiple tumors in the esophagus, hypopharynx, lung, mediastinal and abdominal lymph glands, brain, dermis, and left thumb were confirmed by computed tomography (CT) scans. Biopsy of the esophageal tumor was performed by esophagogastroduodenoscopy. Histopathology confirmed the diagnosis of esophageal squamous cell carcinoma and as the iris tumor was histopathologically similar to the esophageal lesion, the ocular tumor was diagnosed to be a metastatic iris tumor from esophageal cancer (Fig. 2B).

**Figure 3:**
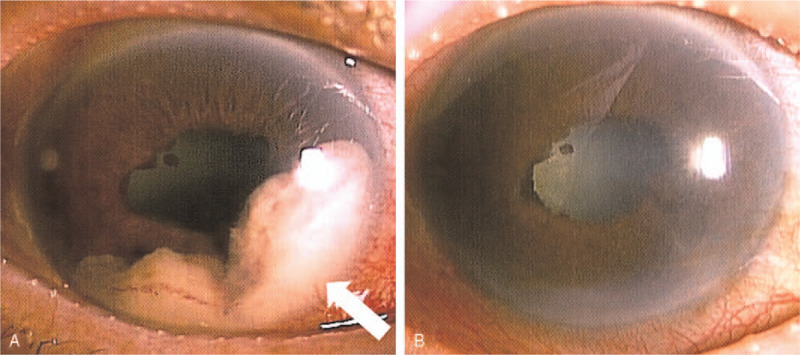
The iris metastasis was diminished after combined therapy. (A) An image of the anterior segment of the right eye. In the anterior chamber, whitish masses with neovascularization located at 9 o’clock and from 2 to 8 o’clock (arrow) were accompanied with posterior synechiae (Three and a half months after initial treatment). (B) An image of the anterior segment of the right eye after chemotherapy and radiation therapy. Therapy effectively diminished iris tumor volume (arrow), except for posterior synechiae (6.5 after initial treatment).

The patient was referred to our hospital for radiation therapy for the brain metastasis because his brain metastasis caused incomplete right hemiparesis, right hemianopsia, and Gerstmann syndrome which is characterized by right hemispatial agnosia, finger agnosia, acalculia, and agraphia. He visited the ophthalmology department 3.5 months after initial treatment. BCVA was 20/400 (OD) and 20/30 (OS) at the visit. IOP of the right eye was 40 mmHg, and the left eye was within the normal range. Slit-lamp examination detected conjunctival hyperemia, slight flare in the anterior chamber, yellowish-white tumors with neovascularization located at 9 o’clock and from 2 to 8 o’clock in the anterior chamber in his right eye. Iris neovascularization, posterior iris synechia, and 360° goniosynechiae were also observed (Fig. 3A). In addition, the fundus was not visible because of corneal epithelial edema, cataract, and vitreous opacity. Ultrasonography showed no tumor in the retina or choroid. Goldman perimeter showed inferior nasal quadrantanopia in the left eye, and a small central visual field in the right eye which could have been caused by the brain metastasis to the left occipital lobe.

Hypotensive eye drops and oral Acetazolamide (Diamox Sanwa Kagaku Kenkyusho Co., Ltd., Japan) were prescribed. The patient received radiation therapy (total 40 Gray/20 fractions) for the iris metastasis. The patient also began systemic chemotherapy with fluorouracil (5-FU Injection, Kyowa Kirin Co., Ltd., Japan) and Cisplatin (Briplatin Injection, Bristol-Myers Squibb Company, NY) for primary cancer concurrently. The metastasis in the iris markedly regressed (Fig. 3B), but IOP of the right eye remained elevated at 25 to 45 mmHg. The patient's right eye lost all light perception 6 months after initial treatment. Although the primary tumor in the esophagus regressed after chemotherapy, the patient died from multiple metastases to the brain and the lung nine months after initial treatment.

## 3 Discussion

According to previous reports, metastatic tumors to the iris originate primarily from malignancies in the breast, lung, and skin (melanoma).^[6]^ Shields et al^[7]^ reported that of 40 patients with metastatic iris tumors, and only 1 (2%) originated from primary esophageal cancer. There have been 8 cases of metastatic tumor to the iris from esophageal cancer reported in 2 decades. Four cases were metastasized from adenocarcinoma^[8–10]^ and 4 were from squamous cell carcinoma,^[11–14]^ and the one had no pathological examination report.^[9]^ Thus, it is extremely rare for esophageal squamous cell carcinoma to metastasize to the iris.

It is often difficult to diagnose metastatic iris tumors in the early stages, and such cases are often misdiagnosed as idiopathic uveitis, because the initial presentation is often pseudo-hypopyon and keratic precipitates.^[6]^ Shields et al reported that there was no history of a primary cancer in 13 of 40 patients (32%) with metastatic iris tumor.^[7]^ We believe that early biopsy of the iris tumor is important if steroid therapy is ineffective in treating putative idiopathic uveitis. The effectiveness of early biopsy is highlighted in the study where squamous cell carcinoma was diagnosed after obtaining a specimen using FNAB of the tumor.^[5]^

The symptoms in our case were initially hyperemia and foreign body sensation. The patient also exhibited vitreous opacity, decreased visual acuity, and secondary glaucoma as the stages of the disease advanced. The primary symptoms of reported metastatic iris tumor were decreased visual acuity, hyperemia, and ocular pain.^[3,7]^ In addition, other reports showed clinical findings such as iris tumor, iridocyclitis, secondary glaucoma, and hyphema caused by neovascular vessels.^[7,8,12]^ Shields et. al reported that visual acuity was 20/40 or better in 27 of the 40 eyes (68%) affected with metastatic iris tumor. In contrast, the patients with worse visual acuity generally had secondary glaucoma as a result of goniosynechiae with tumor in the anterior chamber or retinal detachment secondary to a choroidal metastasis.^[7]^ The visual acuity (OD) in this current case was 20/50 when the iris tumor was diagnosed. However, the delayed commencement of therapy exacerbated the secondary glaucoma which ultimately led to the loss of vision.

There are several described mechanisms leading to secondary glaucoma after metastasis of iris tumors: obstruction of the anterior chamber angle by the neoplasm; invasion of neoplasm to the trabecular meshwork^[15]^; occlusion by cells shed from tumor on the surface of the trabecular meshwork^[16]^; onset of secondary iridocyclitis; and finally, neovascular glaucoma.^[17–19]^ We think both iris neovascularization and angle closure caused increased intraocular pressure in our case, as both tumors were located partially in the anterior chamber and there was also iris neovascularization. Furthermore, ocular pressure did not improve with treatment due to the presence of goniosynechiae, even when the tumors had regressed.

In this case report, the iris tumor markedly regressed after local radiation and concurrent systemic chemotherapy without enucleating the eye. As previously reported, treatment options for metastatic eye tumor include radiation, laser, chemotherapy, anti-vascular endothelial growth factor and enucleation of the eye.^[20]^ If the patient had intolerable ocular pain caused by high intraocular pressure or with phthisis bulbi, enucleation would have been considered. However, local radiation and systemic chemotherapy were successful enough to control ocular pain. Shields et al^[6]^ reported the median period before death after metastasizing to the iris was ten months in 85 deceased patients. The patient in this study died nine and a half months after the onset of the metastatic iris tumor due to multiple systemic metastases.

Using FNAB, we diagnosed a rare iris tumor metastasized from an esophageal squamous cell carcinoma with no concurrent systemic symptoms at the time of diagnosis. Although systemic chemotherapy and radiation therapy were highly effective in reducing the volume of the metastatic iris tumor, the patient died after a brief period. As the prognosis of patients with metastatic iris tumors is poor, it is important for ophthalmologists to bear in mind such diagnoses and conduct systemic investigations when necessary even when there are no concurrent systemic symptoms. Early diagnosis may also minimize ocular symptoms such as pain and also limit negative effects on visual function.

## Author contributions

**Conceptualization:** Hiroko Ozawa, Yoshihiko Usui, and Yohei Tomita.

**Formal analysis:** Hiroko Ozawa.

**Funding acquisition:** Yohei Tomita.

**Investigation:** Hiroko Ozawa and Yoshihiko Usui.

**Project administration:** Yohei Tomita.

**Resources:** Hiroko Ozawa, Yoshihiko Usui, and Tohru Kuribayashi.

**Supervision:** Kazuo Tsubota and Yohei Tomita.

**Writing – original draft:** Hiroko Ozawa, Yoji Takano, and Yohei Tomita.

**Writing – review& editing:** Yoshihiko Usui, Naoki Horiuchi, Tohru Kuribayashi, Toshihide Kurihara, Lois E.H. Smith, and Kazuo Tsubota, Yohei Tomita.

## Supplementary Material

Supplemental Digital Content
